# Supplementary material for: End Sequence Analysis Toolkit (ESAT) expands the extractable information from single-cell RNA-seq data
Source: Genome Res. 2016 Oct;26(10):1397–410. doi: 10.1101/gr.207902.116 (PMC5052061; doi:10.1101/gr.207902.116)
Supplement: Supplemental Material [file supp_gr.207902.116_Supp_Materials.tar.gz › ESAT-esat_v0.1/doc/html/classbroad_1_1pda_1_1seq_1_1protection_1_1_batched_multi_sample_scan_peak_caller.html]

ESAT: broad.pda.seq.protection.BatchedMultiSampleScanPeakCaller Class Reference


ESAT


- Main Page
- Related Pages
- Packages
- Classes
- Files

- Class List
- Class Index
- Class Hierarchy
- Class Members

All Classes Namespaces Files Functions Variables Enumerator Pages

- broad
- pda
- seq
- protection
- BatchedMultiSampleScanPeakCaller

Static Public Member Functions |
List of all members

broad.pda.seq.protection.BatchedMultiSampleScanPeakCaller Class Reference

Inheritance diagram for broad.pda.seq.protection.BatchedMultiSampleScanPeakCaller:

[legend]

Collaboration diagram for broad.pda.seq.protection.BatchedMultiSampleScanPeakCaller:

[legend]

|  |  |
| --- | --- |
| Static Public Member Functions | |
| static String[] | extendSuperArgsForSampleAndChr (String[] commandArgs, String sampleName, String chrName) |
|  | |
| static void | main (String[] args) throws IOException |
|  | |
| Static Public Member Functions inherited from broad.pda.seq.protection.MultiSampleScanPeakCaller | |
| static void | main (String[] args) throws IOException, InterruptedException, DrmaaException |
|  | |

|  |  |
| --- | --- |
| Additional Inherited Members | |
| Public Member Functions inherited from broad.pda.seq.protection.MultiSampleScanPeakCaller | |
|  | MultiSampleScanPeakCaller (String sampleListFile, String bedFile, String chrSizeFile, int window, int step) throws IOException |
|  | |
| void | setFilterByStrand (boolean useStrandFilter) |
|  | |
| void | setExtraFields (boolean useExtraFields) |
|  | |
| void | setBinomialScore (boolean binomialScore) |
|  | |
| void | setPeakCutoffMostCommonReplicate (double maxPct) |
|  | |
| void | setPeakTrimQuantile (double trimPeakQuantile) |
|  | |
| void | setPeakWindowCountCutoff (double peakCountCutoff) |
|  | |
| void | setPeakWindowScanPvalCutoff (double peakScanPvalCutoff) |
|  | |
| void | setFirstReadTranscriptionStrand (boolean firstReadIsTranscriptionStrand) |
|  | |
| void | setExpressionScanPvalueCutoff (double expressionScanPvalCutoff) |
|  | |
|  | MultiSampleScanPeakCaller (String expressionBamFile, String signalBamFile, String bedFile, String chrSizeFile) throws IOException |
|  | |
|  | MultiSampleScanPeakCaller (String expressionBamFile, String signalBamFile, String bedFile, String chrSizeFile, int window, int step) throws IOException |
|  | |
| boolean | isExpressedInAllControlSamples (Gene gene) |
|  | |
| boolean | isExpressed (Gene gene) |
|  | |
| Collection< Gene > | getSingleSampleScanPeaks (SampleData sample, Gene gene) throws IOException |
|  | |
| Collection< Annotation > | mergePeaks (Collection< Annotation > peaks) |
|  | |
| void | setCoordinateSpace (CoordinateSpace space) |
|  | |
| CoordinateSpace | getCoordinateSpace () |
|  | |
| Protected Member Functions inherited from broad.pda.seq.protection.MultiSampleScanPeakCaller | |
|  | MultiSampleScanPeakCaller (MultiSampleScanPeakCaller other) throws IOException |
|  | |
| void | initializeFilterRejectWriters (String commonSuffix, String outDir) throws IOException |
|  | |
| void | closeFilterRejectWriters () throws IOException |
|  | |
| String | getPeakBedFileName (SampleData sample, String outDir, String chrName) |
|  | |
| void | writeSingleSampleScanPeaks (SampleData sample, String outFile, String chrName) throws IOException |
|  | |
| SamplePermutation | getOneRandomSamplePermutation () |
|  | |
| void | setLoggerLevel (Level level) |
|  | |
| Static Protected Member Functions inherited from broad.pda.seq.protection.MultiSampleScanPeakCaller | |
| static MultiSampleScanPeakCaller | createFromCommandArgs (String[] commandArgs) throws IOException |
|  | |
| static boolean | commandLineHasDebugFlag (String[] commandArgs) |
|  | |
| static String | commandLineOutDir (String[] commandArgs) |
|  | |
| Protected Attributes inherited from broad.pda.seq.protection.MultiSampleScanPeakCaller | |
| Map< String, Collection< Gene > > | genes |
|  | |
| Map< SampleData, Map< Gene,   Map< Annotation,   BinomialEnrichmentScore > > > | binomialWindowScores |
|  | |
| GenomeSpaceSampleData | expressionData |
|  | |
| ArrayList< SampleData > | controlSamples |
|  | |
| ArrayList< SampleData > | signalSamples |
|  | |
| ArrayList< SampleData > | allSamples |
|  | |
| int | windowSize |
|  | |
| int | stepSize |
|  | |
| int | numControls |
|  | |
| int | numSignals |
|  | |
| int | numSamples |
|  | |
| Random | random |
|  | |
| String | sizeFile |
|  | |
| Static Protected Attributes inherited from broad.pda.seq.protection.MultiSampleScanPeakCaller | |
| static Logger | logger = Logger.getLogger(MultiSampleScanPeakCaller.class.getName()) |
|  | |
| static String | FILTER\_REJECT\_DIR = "filter\_rejects" |
|  | |

## Detailed Description

Author
:   prussell

## Member Function Documentation

|  |  |  |  |
| --- | --- | --- | --- |
| static String [] broad.pda.seq.protection.BatchedMultiSampleScanPeakCaller.extendSuperArgsForSampleAndChr | ( | String[] | *commandArgs*, |
|  |  | String | *sampleName*, |
|  |  | String | *chrName* |
|  | ) |  |  |

static

|  |  |  |  |  |  |
| --- | --- | --- | --- | --- | --- |
| static void broad.pda.seq.protection.BatchedMultiSampleScanPeakCaller.main | ( | String[] | *args* | ) | throws IOException |

static

Parameters
:   |  |  |
    | --- | --- |
    | args |  |

Exceptions
:   |  |  |
    | --- | --- |
    | IOException |  |

Here is the call graph for this function:

---

The documentation for this class was generated from the following file:

- java/broad/pda/seq/protection/BatchedMultiSampleScanPeakCaller.java


---

Generated on Fri Aug 15 2014 15:11:02 for ESAT by  

 1.8.7
